# Technical note: contour plot visualization of the light adapted electroretinogram using a generalized additive model

**DOI:** 10.1007/s10633-025-10078-3

**Published:** 2026-01-18

**Authors:** Marek Brabec, Lynne Loh, Irene O. Lee, Fernando Marmolejo-Ramos, David H. Skuse, Dorothy A. Thompson, Paul A. Constable

**Affiliations:** 1 Institute of Computer Science of the Czech Academy of Sciences, Pod Vodarenskou Vezi 2, 182 00 Prague 8, Czech Republic; 2 National Institute of Public Health, Srobarova 48, 100 00 Prague 10, Czech Republic; 3 Caring Futures Institute, College of Nursing and Health Sciences, Flinders University, Adelaide, 5000 Australia; 4 Behavioural and Brain Sciences Unit, Population Policy and Practice Programme, UCL Great Ormond Street Institute of Child Health, University College London, London, UK; 5 College of Education, Psychology and Social Work, Flinders University, , Adelaide, Australia; 6 The Tony Kriss Visual Electrophysiology Unit, Clinical and Academic Department of Ophthalmology, Great Ormond Street Hospital for Children NHS Trust, London, UK; 7 UCL Great Ormond Street Institute of Child Health, University College London, London, UK

**Keywords:** Tensor spline, Surface, Electroretinogram, Response series

## Abstract

**Purpose:**

To describe a new method to visualize the amplitude profile of light adapted ERGs as a series of contour plots in response to a Flash Strength series.

**Methods:**

Light adapted ERGs from a dataset of *n* = 88 Autism Spectrum Disorder (ASD) and* n* = 70 typically developing Control participants were used incorporating 10 flash strengths ranging from 12 to 446 Troland seconds (Td.s). The region chosen for analysis included the baseline, a-wave and ascending limb of the b-wave and included *n* = 1736 control and *n* = 1300 ASD waveforms. A Generalized Additive Model (GAM) with complexity penalized tensor product splines was applied to the waveform intervals.

**Results:**

Contour plots derived from the ASD and Control groups revealed a pattern of reduced amplitude in the ascending limb of the b-wave for the ASD group. Representative responses were derived from the GAM model for discrete Flash Strengths to exemplify the changing profile of the ERG waveform. A contour plot of the derived z-scores provided a qualitative evaluation of the differences in the mean amplitudes which was concentrated on the b-wave.

**Conclusions:**

The production of contour plots based on amplitude, time and Flash Strength with minimal a priori assumptions provide an additional approach to analyzing stimulus response data series and may support clinical applications. Additionally, the GAM-based methodology provides a tool for simultaneous investigation of the amplitude changes over time and Flash Strength which can be useful for theoretical purposes.

**Supplementary Information:**

The online version contains supplementary material available at 10.1007/s10633-025-10078-3.

## Background

Analysis of the ERG a-wave typically involves mathematical modelling of the rod and cone a-wave families of responses to a sequence of Flash Strengths (FSs) [1]. The slope of the initial descending portion of the a-wave relates to the rate of phototransduction [2, 3] and state of adaptation within the photoreceptors [4]. Several models have been developed based on exponential fits to the descending portion of the a-wave under dark- and light-adapted conditions as a function of FS [5]. Modelling of the dark-adapted a-wave has also been performed using electrical circuits to reflect the physiological generators [6]. ISCEV has published an extended protocol to determine the cone contribution to the dark-adapted a-wave [7]. These all provide possible methods to explore the a-wave under dark, and light adapted conditions.

The light-adapted a-wave is shaped by post receptoral neurons as demonstrated through blockade of cone-horizontal and cone-hyperpolarizing bipolar cells in macaque [8]. In rat the OFF-pathway contributes to the ascending portion of the dark-adapted a-wave [9] and may alter the dark current of the rod photoreceptors [10]. The ascending portion of the a-wave is shaped by the intrusion of the bipolar cells that contribute to PII as described by Granit with the slow fast PIII representing the hyperpolarization of the photoreceptors [11]. Thus, the shape of the a-wave and its description give insights into the phototransduction cascade of the photoreceptors, and the contribution of post receptoral neurons (horizontal cells and bipolar cells) as well as the contribution of the PII components from the bipolar and glial cells [12–14]. The dark-adapted b-wave is typically modelled as a luminance response series using the Naka-Rushton function [15]. The light adapted (LA) b-wave amplitude can be modelled using mathematical functions that describe the photopic hill with the rise and plateauing of the b-wave amplitude at high FSs [16].

To provide a new visualization of the changing shape of the a-wave and ascending portion of the b-wave, with minimal preconceived assumptions, this report used a Generalized Additive Model (GAM) where the ERG waveform ‘signal’ was treated as the response and the explanatory variables were time (*t*) and (FS). The main features of the model were: i) it was nonparametric and hence it did not enforce any a priori functional form (e.g. exponential) with respect to *t* or FS, ii) it allowed for the interaction of the two continuous explanatory variables (*t* and FS) and their influence on the ERG signal amplitude. In other words, the model allowed for deformation of the ERG amplitude trajectory with FS in a way that was fully dictated by the empirical raw data. This was achieved by employing a (complexity penalized) tensor product spline in *t* and FS.

This approach was built on a previous, simpler analysis using GAM, in which there were two additive functions of one variable [17]. Here, we modelled the initial portion of the ERG waveform with a function (*f*) of two variables (time, (*t*) and (FS)) at once. The main purpose was to allow for the interaction of *t* and FS on their influence on the ERG signal amplitude. This interaction can be described as the deformation of the ERG time trajectory with FS. This view allows for a deeper analysis of the surface and features of the ERG waveform that are represented as a series of contour lines corresponding to the mean amplitude of the ERG waveforms as FS and *t* change. This visualization is similar to the density plots reported for the mfERG [18] but in this case time and FS are on the ordinate axes with the amplitude plotted as a surface map defined by contour lines.

This report demonstrates the use of the smooth function *f*(*t*,FS) plotted as an object to express the ERG waveform profile deformation with both FS and *t*, rather than plotting conventional amplitude vs time for each FS as a family of responses. The initial portion of the ERG waveform was chosen in this instance as an example of the method, but the analysis could be extended across the full waveform to explore for example the i-wave, photopic negative response and photopic hill.

## Methods

The ERG waveforms used for purposes of this report had been recorded as part of a prior study with the details of acquisition described in detail previously [19, 20]. In brief, light-adapted ERGs were recorded using the RETeval (LKC Technologies, Gaithersburg, MD) with a randomized flash series of 9 strengths from 12, 21, 35, 70, 113, 178, 251, 356 and 446 Troland seconds (Td.s) on a 1130 Td white background. Included in the series was the 85 Td.s on an 840 Td white background that was recorded after the nine randomized strength series and is equivalent to the LA3 ISCEV standard flash in the right then left eye of each participant. Signals were filtered 0.1–300 Hz and 30–60 and averages were obtained at each FS to produce the signal average. Waveforms were excluded from the average if they fell outside of 2 SD of the averaged signal to exclude artefacts caused by eye movements or blinks. The interval of interest was defined based on the first 100 rows of sampled data. Owing to slight variations in sampling rate of ~ 2 kHz, the data extended for the ASD group from − 20.51110 to 31.19921 ms, and for the Control group from − 20.51190 to 31.19867 ms which captured the a-wave and the ascending limb of the b-wave for the FS series. Table 1 shows the number of ERG waveform samples for each FS used (*n* = 1300 ASD and *n* = 1736 Control) to model the *f*(*t,*FS). The LA 3 ISCEV Standard ERG specifies a dilated pupil with 3 cd.s.m^−1^ flash on a 30 cd.m^−1^ background. These Troland values correct for the participants pupil size in real time [21].

**Table 1:** Number of individual flash strengths from both eyes used to construct the contour plots

Flash strength (Td.s)	Control	ASD
12	174	121
21	172	130
35	175	130
70	178	132
85	157	118
113	177	134
178	175	137
251	175	132
356	175	132
446	178	134
Total	1736	1300

### Participants

The participants were individuals with a diagnosis of autism spectrum disorder (ASD) (*n* = 70) and typically developing individuals grouped as the Control group (*n* = 88). The ASD group were aged (mean ± SD) 12.9 ± 4.3 years (range 5.9–27.3 years) with 56 male and 14 female participants. The Control group were aged 13.3 ± 4.9 years (range 5.0–26.7 years) with 46 male and 42 female participants.

The parent/guardian, or if the participant was aged over 16 years gave written informed consent before testing. The study was approved by the South East Scotland Research Ethics Committee UK and the Human and Research Ethics Committee, Flinders University. All participants gave written informed consent or their parent/guardian with the study conforming to the Declaration of Helsinki.

### Generalized additive model

The following GAM, [22] with complexity penalized tensor product splines [23] was used and is shown in Eq. 1.

1 $${Y}_{ijk}={\beta }_{0}+{b}_{i}+f\left({t}_{ijk},{FS}_{ij}\right)+{\varepsilon }_{ijk}$$

where:

- ${Y}_{ijk}$is the ERG signal for the *i*-th individual, *j*-th level of FS and *k*-th *t* (time points generally differed slightly among individuals).
- ${t}_{ijk}$ is the *k*-th time point for the *i*-th individual and *j*-th level of FS.
- ${FS}_{ij}$ is the FS for the *i*-th individual and *j*-th level of FS.
- ${\beta }_{0}$ is an unknown coefficient (intercept) to be estimated from the data.
- ${b}_{i}$ is a random individual (eye) effect. We assumed ${b}_{i}\sim N\left(0,{\sigma }_{b}^{2}\right)$, independently across eyes.
- ${\varepsilon }_{ijk}$ is a random within-individual error term. The Gaussian working model, i.e. ${\varepsilon }_{ijk}\sim N\left(0,{\sigma }^{2}\right)$, was assumed independently across individuals and time points.
- $f\left(.,.\right)$ is a smooth unknown function of two variables (*t* and FS) to be estimated non-parametrically from the data. In our model, it is implemented as a tensor product spline penalized for complexity.

The model was identified (i.e. its unknown scalar and functional parameters together with variance estimated) via optimization of the penalized likelihood [22]. The penalty coefficients were obtained via generalized cross validation optimization [24, 25]. The mgcv library [22] [19] and R [26] were used for computations. In this example the right and left eye recordings were combined for each individual but in principle either eye or a random eye could be used (we have done these analyses without profound changes in the results). In this case the larger number of samples improved precision of the modeling and visualization of the function *f*(*t*,FS) that was built from the ERGs recorded in each eye from the participants (while we used the person-specific random effect to prevent the pseudoreplication phenomenon [27].

## Results

**Fig. 1:**
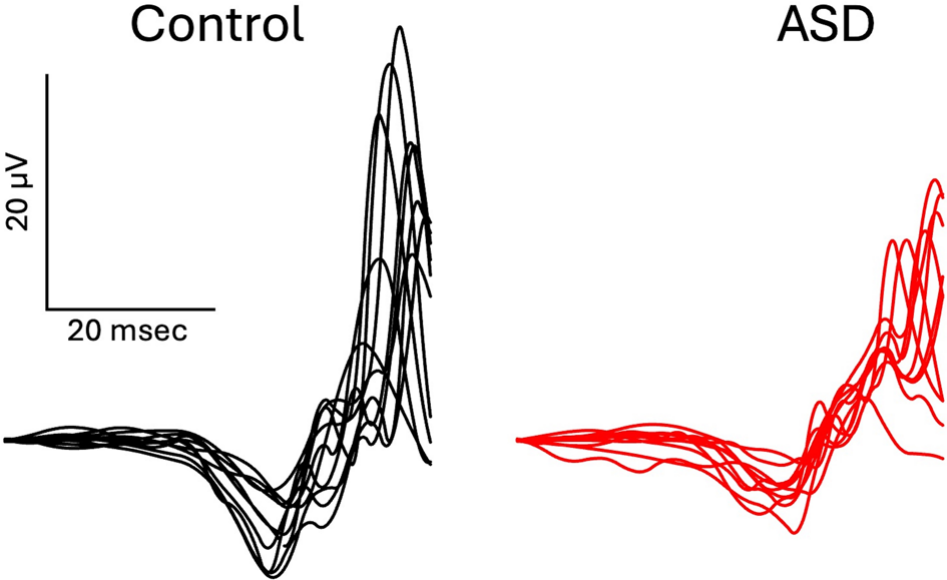
Representative ERG waveforms from a Control and an ASD participant. The response series was derived from Flash Strengths ranging from 12 to 446 Td.s. Note the generally lower amplitude of the ERG in the ASD participant in this case

ERG waveforms were pooled from the right and left eyes to generate the contour plots, although responses were similar with either left or right eye and we have previously shown that either eye is representative of the group [17, 28]. Figure 1 illustrates the family of ERG waveforms from a representative Control and ASD participant.

**Fig. 2:**
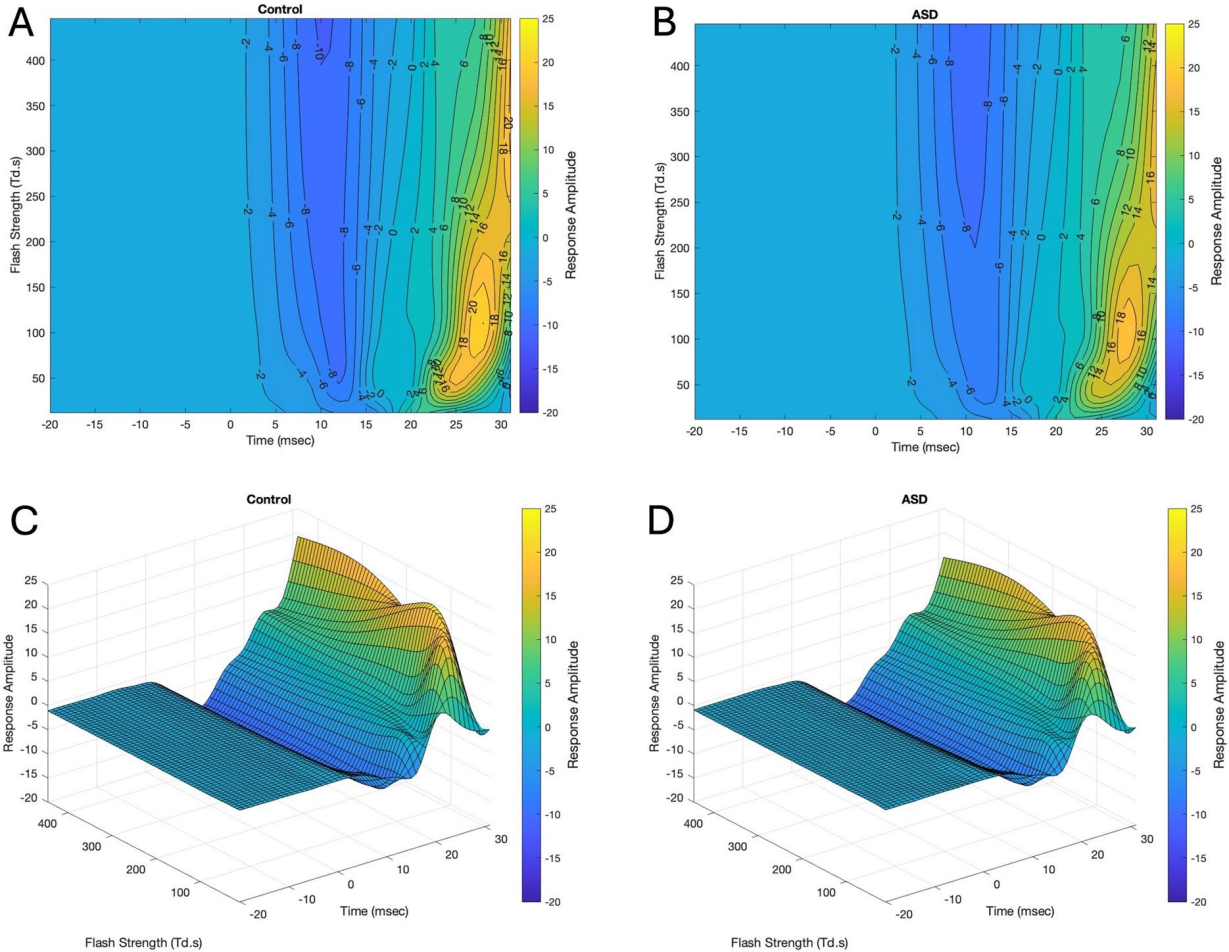
Contour plots of the functions *f*(*t*,FS) of the baseline, a-wave and ascending portion of the b-wave of the light adapted ERGs for the Control (**A**) and ASD (**B**) groups. The numerical values on each contour line represent the mean value of the function. The surface representation shows a minimum (blue) corresponding to the a-wave minima at ~ 12 ms with the peak amplitude (yellow) occurring at ~ 28 ms on the b-wave encompassing the OPs. Perspective views of the surfaces are shown for the Control (**C**) and ASD (**D**)

Figure 2 illustrates the contour and surface plots of the function *f*(*t*, FS) for the ASD and Control groups which are displayed with the contour lines representing the mean response amplitude as a function of time and FS. The blue shades represent negative amplitudes corresponding to the a-wave with the lighter tallow shades the positive amplitude values associated with the b-wave. MATLAB rotational views are available as supplementary files for the ASD and Control surface plots: (ASD 3d.mat and Control 3d.mat). In addition, the MATLAB code and dataset are available to generate the figures in this report (plot3dContour.mat).

**Fig. 3:**
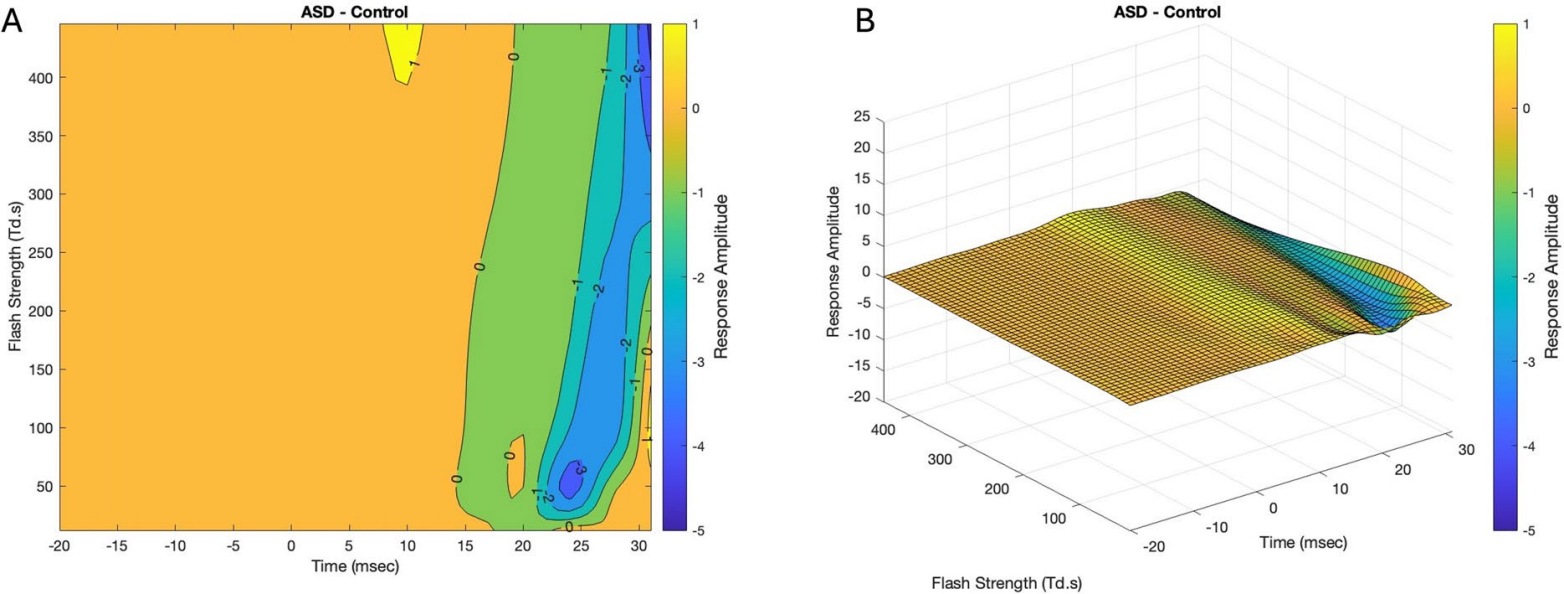
The difference of the contour plots between the ASD and Control ERG waveform regions as a function of time and Flash Strength is shown in (**A**). The darker blue shades represent the greatest difference in amplitude. The profile of the amplitudes is lower for the ASD group (hence a negative difference), and this difference is greatest at the higher flash strengths of ~ > 350 Td.s. The surface plot of the difference is shown in (**B**)

To visualize regions where the ERG amplitude showed systematic differences between the groups a difference plots (contour and surface) for ASD-Control of the *f*(*t*,FS) are shown in Fig. 3. A rotational view image file is provided as supplementary material (difference 3d.mat).

### z-score

**Fig. 4:**
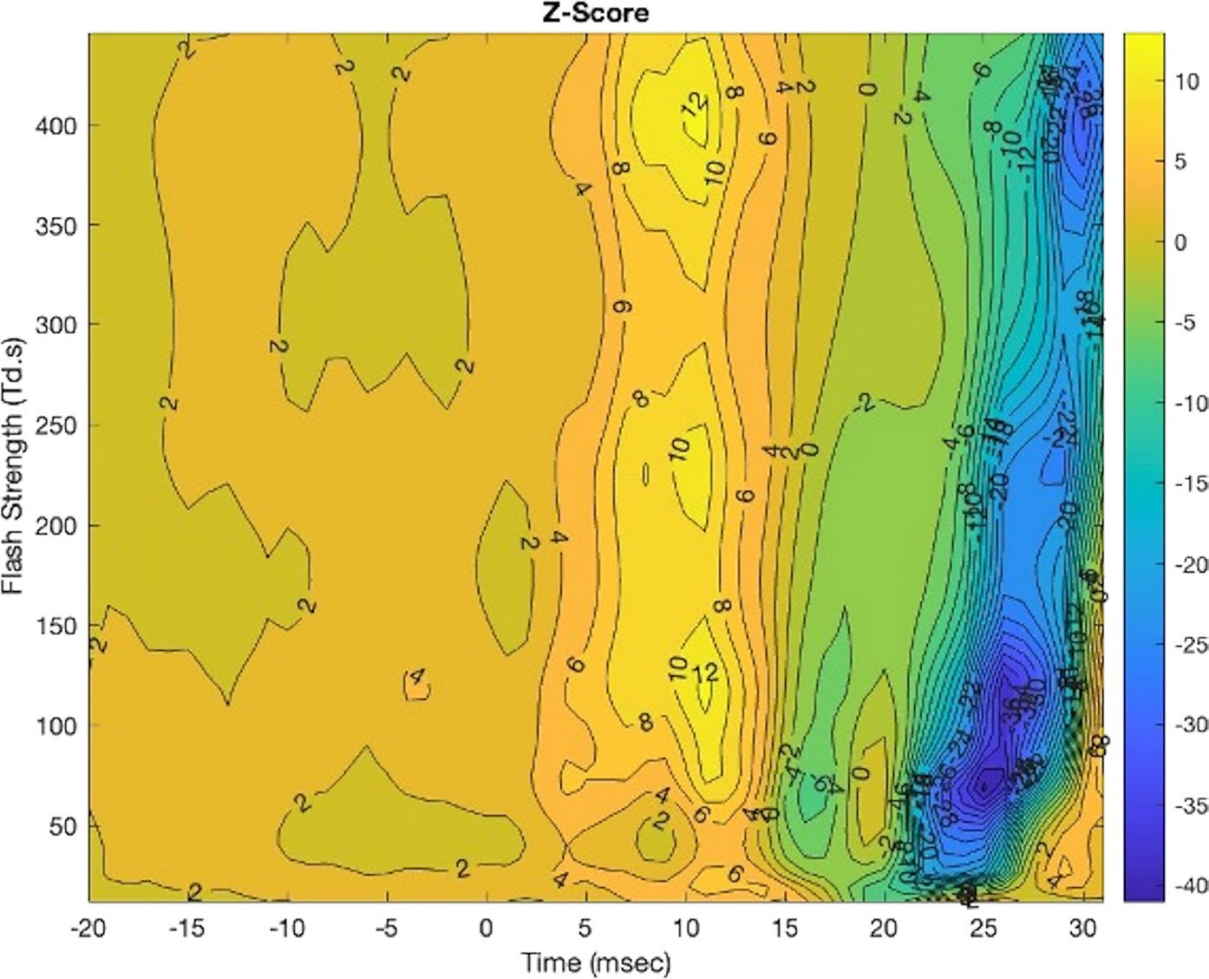
Contour plot of the z-scores for ASD-Control differences between the groups. The main differences between the waveforms are evident between t = 25 and 31 ms at Flash Strengths in the range of 50–100 Td.s

Figure 4 plots the calculated z-scores (ratio of the estimate to its estimated standard error) for ASD-Control as a qualitative measure of the differences that have the greatest disparity between the groups. The z-scores indicated the substantial negative inter-group difference lies between 25–30 ms with FS range of 50–100 Td.s.

### Modelled waveforms

**Fig. 5:**
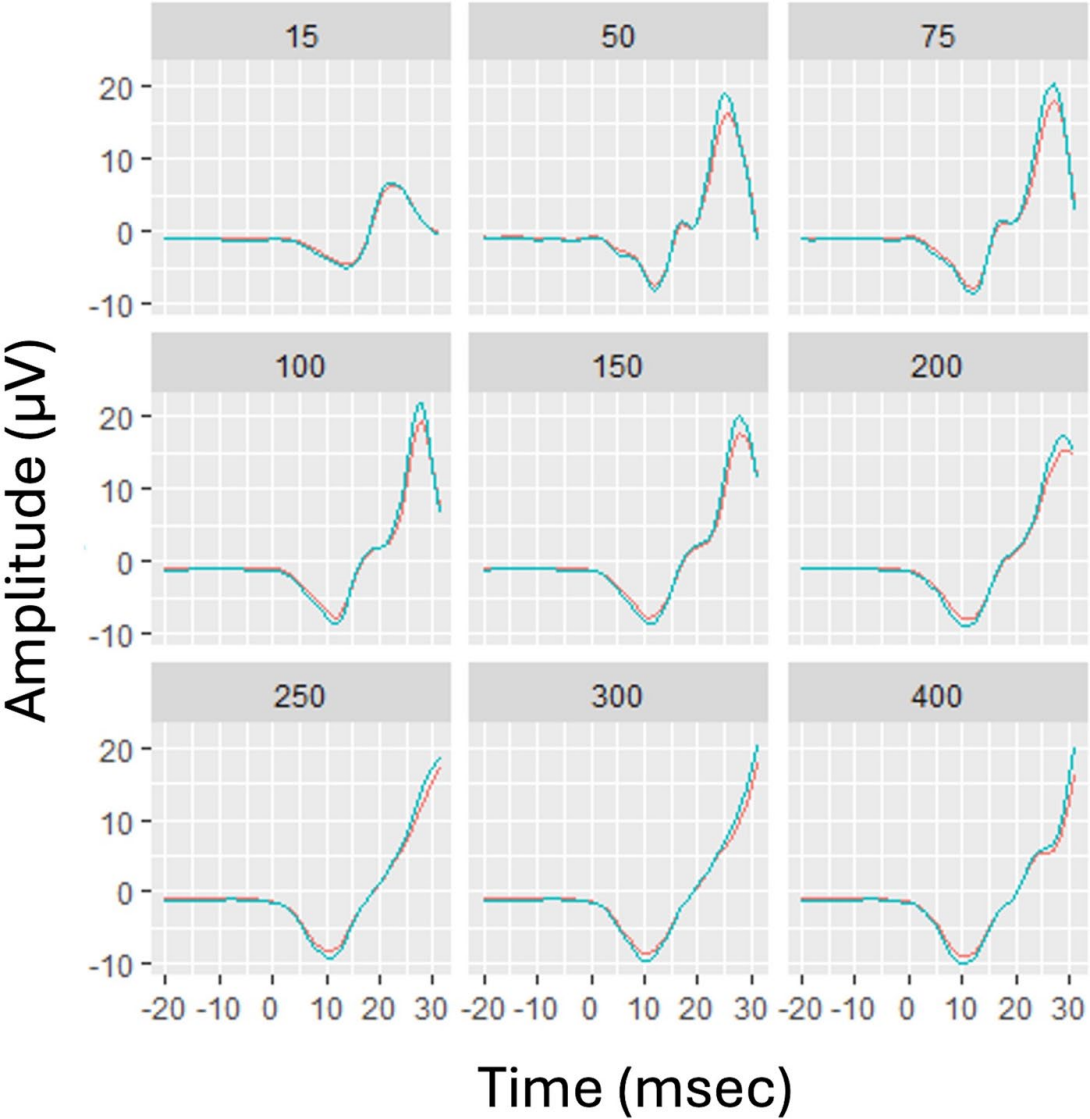
Modelled ERG waveforms derived from the smoothed function *f*(*t*,FS) for the ASD (red) and Control (blue) groups provides a visualization of the changing shapes of the waveforms across determined Flash Strengths (FSs)

Derived from the *smoothed function* of *f*(*t*,FS) the ERG waveform shapes can be computed at FS within the modelled range. Figure 5 shows the results from the two models (for ASD and control fitted on the full data and evaluated at a selection of FSs (15, 50, 75, 100, 150, 200, 250, 300, and 400 Td.s). Figure 5 shows the potential to generate a model of the ERG waveform at any defined FS that lies within the contour plot.

## Discussion

This initial analysis, using a GAM approach to model the early stages of the ERG waveform as a function of FS and *t,* provides an additional way of visualizing the morphological features of the ERG. This approach may offer additional insights into the ERG characteristics recorded as part of a dark-adapted [15] or light-adapted [29] flash series and related clinical waveforms such as the PERG [30] and VEPs [31, 32]. The smoothed function generated using splines also enables the generation of representative waveforms at specified FSs to highlight the main differences between waveforms at defined FSs. The ability to interpolate the shape of the ERG waveform to illustrate differences in the waveforms across a wider range of FSs offers a new perspective for examining changes in the ERG that are not fixed to the main peaks and troughs. The modelling and visualization of the ERG waveform using this approach builds on previous studies that have modelled the ERG luminance response series [2, 3, 5, 10, 16, 33, 34].

Current analyses of luminance response series rely on examining the coefficients of the functions that describe the changes in b-wave amplitude with FS. In the case of the dark-adapted series these are modelled using the Naka-Rushton function with an assumption that *n* = 1, with V_max_ interpreted as being dependent on the number of photoreceptors and the constant K that of ‘retinal sensitivity’ [15]. For the light adapted flash series the ‘photopic hill’ [35] that describes the rise and fall of the b-wave amplitude with FS can be mathematically described by a combination of functions with a logistical and Gaussian component [16] that provide insights into the relative contributions of the retinal ON- and OFF- pathways [34]. These methods rely on the amplitude of the b-wave and FS, whilst this GAM model provides an alternative visualization of the variations in the waveform shape with FS over time.

In contrast to the typical representation of the family of ERG waveforms depicted in Fig. 1, the contour and surface plots illustrated in Fig. 2 provide a surface representation of the smoothed amplitude change across a continuous range of FSs. This visualization enables regions to be identified that differ in amplitude or gradient as depicted by the color scales. In this example, subtracting the plots between groups (ASD-Control) enabled a qualitative evaluation of how the waveforms vary between groups. In the case illustrated in Fig. 3 the a-wave amplitude profile showed no deviations between the groups. In contrast, islands or regions within the contour plots shaded dark blue highlight differences observed for FSs centered on ~ 50 Td.s in the time intervals between 24–26 ms and for FSs centered on ~ 400 Td.s at time 31 ms were evident. The lower amplitudes may be the result of reduced contribution of the OPs as previously reported for this dataset [19, 36, 37]. There was no difference observed in the descending portion of the a-wave indicating the lower amplitudes are not consequent upon differences in phototransduction [3].

Figure 2 illustrates the three-dimensional view that is possible through modelling the ERG with GAMs. The smoothed surface defined by the contour lines provides an impression of the formation of the principal ERG components in this region–namely the a-wave the b-wave and the OPs. Derived from the smoothed function, Fig. 5 then can be constructed to illustrate the main differences in the features of the ERG between the groups. The modelling allows for the visualization of the smoothed ERG at determined FSs. In this example the chosen FSs from 50–200 Td.s indicates a reduction in the b-wave amplitude for the ASD group. As a future application this approach can highlight surfaces of maximal difference which could inform study designs that aim to differentiate disease states and/or anticipate regions of interest for observing outcome effects of interventions.

One limitation of the current modelling is how to quantify the statistical differences between the groups. The main problem is the issue of repeated measurements that arise because of a high number of comparisons at multiple positions which are highly correlated due to the inherent physical/physiological reasons and because of the way the tensor product spline was fitted to the surface. To address this limitation, Fig. 4 provides the contour plot of the z-scores that provides a qualitative visualization of the regions where the greatest differences in the mean differences in amplitude occur.

A future method to extend this work would be to blend the GAM method proposed here and Fourier Analysis Networks (FAN) [38]. FAN is a new type of neural network specifically designed to handle repeating patterns in data while maintaining general-purpose capabilities. We envisage that GAM and FAN could be blended in complementary ways, with FAN capturing periodic patterns in the ERG signal while GAM splines model non-periodic, smooth relationships. Another promising approach would be to use FAN layers to extract periodic features from raw data, then feed these features into a GAM for final prediction. We believe this blend could be particularly powerful for analyzing complex functional data that contains both periodic and non-periodic components, such as ERGs. The combination would leverage the strengths of both methodologies-FAN's ability to model periodicity and GAM's flexibility in capturing smooth relationships-resulting in a more comprehensive analytical framework for ERG signal interpretation. Another possibility is to use GAM (or other types of semiparametric statistical models) for the raw (non-filtered) data to explore spectrum (specific smooth of the results at Fourier frequencies) in detail and to compare the spectra of different groups using Bayesian statistics. The contour plot built in three dimensions using a GAM model provides a new way of visualizing the changing shape of the ERG as part of a stimulus–response series, which builds on previous models using statistical approaches using regularization [28] and Bayesian modelling [17].

This approach may support clinical testing in populations where a surface map may provide insights into variations in amplitude [33], timing [39] and shape [40, 41] of the ERG waveform as a function of time, amplitude and FS. The analysis could be applied to group datasets as in this case or for an individual response to characterize the changes in the ERG’s shape with FS. This work contributes to the expanding field of ERG signal analysis using statistical [17, 28], signal analysis [36, 42–51] or mathematical modelling [5, 10, 15, 16, 29]. This method may allow for an alternative representation of the ERGs signal transformation with FS based on smoothed waveforms derived from the GAM model to illustrate group differences such as the b-wave amplitude as in this case. In conclusion, the representation of the amplitude with time and FS as a continuous surface enables the appreciation of the degree of difference in the ERG trajectory in response to the flash series.

## Supplementary Information

Below is the link to the electronic supplementary material.

Supplementary file1 (XLSX 1873 KB)

Supplementary file2 (FIG 152 KB)

Supplementary file3 (FIG 152 KB)

Supplementary file4 (FIG 157 KB)

Supplementary file5 (MP4 8023 KB)

Supplementary file6 (M 4 KB)

## Data Availability

Dataset is available at: 10.6084/m9.figshare.29345636.v1.

## Code availability

On request from Marek Brabec.
